# DNase inhibits early biofilm formation in *Pseudomonas aeruginosa*- or *Staphylococcus aureus*-induced empyema models

**DOI:** 10.3389/fcimb.2022.917038

**Published:** 2022-10-12

**Authors:** Wusheng Deng, Yanmei Lei, Xiujia Tang, Dingbin Li, Jinhua Liang, Jing Luo, Liuyuan Liu, Wenshu Zhang, Liumei Ye, Jinliang Kong, Ke Wang, Zhaoyan Chen

**Affiliations:** ^1^ Department of Respiratory and Critical Care Medicine, The First Affiliated Hospital of Guangxi Medical University, Nanning, China; ^2^ Department of Orthopedic Trauma and Hand Surgery, The First Affiliated Hospital of Guangxi Medical University, Nanning, China; ^3^ Intensive Care Unit, The First Affiliated Hospital of Guangxi Medical University, Nanning, China

**Keywords:** DNase, *Pseudomonas aeruginosa*, *Staphylococcus aureus*, pleural empyema, biofilm

## Abstract

Anti-infection strategies against pleural empyema include the use of antibiotics and drainage treatments, but bacterial eradication rates remain low. A major challenge is the formation of biofilms in the pleural cavity. DNase has antibiofilm efficacy *in vitro*, and intrapleural therapy with DNase is recommended to treat pleural empyema, but the relevant mechanisms remain limited. Our aim was to investigate whether DNase I inhibit the early biofilm formation in *Pseudomonas aeruginosa*- or *Staphylococcus aureus*-induced empyema models. We used various assays, such as crystal violet staining, confocal laser scanning microscopy (CLSM) analysis, peptide nucleic acid-fluorescence *in situ* hybridization (PNA-FISH), and scanning electron microscopy (SEM) analysis. Our results suggested that DNase I significantly inhibited early biofilm formation in a dose-dependent manner, without affecting the growth of *P. aeruginosa* or *S. aureus in vitro*. CLSM analysis confirmed that DNase I decreased the biomass and thickness of both bacterial biofilms. The PNA-FISH and SEM analyses also revealed that DNase I inhibited early (24h) biofilm formation in two empyema models. Thus, the results indicated that DNase inhibited early (24h) biofilm formation in *P. aeruginosa*- or *S. aureus*-induced rabbit empyema models and showed its therapeutic potential against empyema biofilms.

## Introduction

Pleural empyema is a serious infectious condition of the pleural cavity associated with high morbidity and mortality (Lehtomaki et al., 2020a; Lehtomaki et al., 2020b). There are approximately 80,000 annual cases of pleural infections in the USA and UK combined (Idell et al., 2017). Despite optimal management, outcomes remain poor with a mortality rate of 20% in adults (Elsayed et al., 2018) and 30% in older people (Bedawi et al., 2021). Approximately 80% of pleural infections can be cured with antibiotics and through chest drainage, and the use of combination therapy with a fibrinolytic agent and DNase reduces the frequency of surgical referrals (Rahman et al., 2011); however, 16–27% of the patients treated in this manner end up requiring surgical treatment (Sundaralingam et al., 2021). Antibiotic resistance and biofilm formation are the main challenges encountered when treating pleural empyema.

Biofilms are sessile communities of microbes surrounded by a self-produced polymer matrix (Rybtke et al., 2015). The extracellular polymeric substances (EPS) that form the matrix comprise polysaccharides, proteins, lipids, and extracellular DNA (eDNA). eDNA contributes to the structural integrity of the biofilm matrix, which is essential for bacterial attachment and aggregation in the early stages of biofilm formation (Davide et al., 2021; Panlilio and Rice, 2021). Approximately 80% of bacterial infections are related to biofilms (KaiLing et al., 2022), which are difficult to remove completely. *P. aeruginosa* and *S. aureus* are the most common pathogens in pleural empyema (Bedawi et al., 2019; Hassan et al., 2019; Chen et al., 2021), and *P. aeruginosa* can form biofilms in pleural empyema (Zhang et al., 2020). Microbial cells within biofilms are 10–1000 times more resistant to antibiotics than planktonic bacteria (Sharma et al., 2019). Biofilm-related infections are recalcitrant to clearance by antimicrobials and host defense molecules (Rather et al., 2021), resulting in antibiotic-resistant and chronic infections. Inhibiting biofilm formation is an effective approach to improve antibacterial efforts and increase the susceptibility of pathogens to antimicrobials.

DNase is an endonuclease that cleaves phosphodiester bonds adjacent to pyrimidines to produce polynucleotides with terminal 5ʹ-phosphates. DNase is used to remove DNA from protein and nucleic acid samples (Karygianni et al., 2020). Moreover, DNase promotes the antibiotic susceptibility of biofilm-associated pathogens in infections (Tetz et al., 2009). DNase degrades eDNA in the matrix, weakening it and facilitating antimicrobial diffusion. Studies have shown that DNase can inhibit or degrade biofilms *in vitro* (Farisa Banu et al., 2017; Ramaraj et al., 2020; Srikanth et al., 2021), and possession of DNase coating increases the antimicrobial activity of *S. aureus* biofilm therapy (Meraj et al., 2020). However, whether DNase inhibits biofilm formation in pleural empyema remains unknown.

Treatment with fibrinolytic agents and DNase is recommended for patients with empyema when intrapleural therapy is being considered (Chaddha et al., 2021). The Multicenter Intrapleural Sepsis Trials (MIST2) (Rahman et al., 2011) results indicated that intrapleural t-PA/DNase therapy improved fluid drainage and reduced the frequency of surgical referral in patients with pleural infection, but the relevant mechanisms remain unclear. In a consensus statement about DNase and fibrinolytic treatments of pleural empyema, it was hypothesized that the deterioration in patients treated with DNase monotherapy may be due to biofilm disruption mediated by DNase in the pleural cavity (Chaddha et al., 2021). A study showed that the combination with DNase therapy was effective against biofilm-associated wound infections and accelerated wound healing (Kumar et al., 2019). Hence, we speculate that DNase can inhibit biofilm formation in pleural empyema.

In this study, we first demonstrated that DNase I inhibited early biofilm formation in *P. aeruginosa* and *S. aureus in vitro*. Next, we established rabbit empyema models induced by *P. aeruginosa* or *S. aureus*, and investigated the inhibitory effects of DNase I on early biofilm formation.

## Materials and methods

### Bacterial strains and growth conditions

Both *P. aeruginosa* PAO1 and *S. aureus* (ATCC25913) were stored in Luria–Bertani (LB) broth containing 25% glycerol at –80°C. The *P. aeruginosa* was grown in LB, and *S. aureus* was grown in tryptic soy broth (TSB) supplemented with 0.5% glucose. The bacterial suspensions were standardized to an optical density (OD) of 0.1 at 600 nm (Chen et al., 2016). We prepared dilutions of 2 mL LB or TSB with 10^8^ colony-forming units (CFU)/mL bacteria [OD = 0.1], and then serially diluted them to 10^6^ CFU/mL for further experiments (Zhang et al., 2020).

### *In vitro* experiments

#### Growth curve

We used a previously described method (Luo et al., 2016) to compare growth curves. Briefly, we obtained overnight cultures of bacterial solutions (OD_600_ = 0.1), and diluted them with sterile phosphate-buffered saline (PBS) to achieve cell suspensions at OD_600_ = 0.05. The suspensions were supplemented with different concentrations of DNase I (1000 U; Baoriyi Biotechnology, Beijing, China) at 200, 100, 50, 25, 12.5, 6.25, and 0 U/mL. DNase I was derived from a nonanimal host, had an activity of 5 U/μL, and was stored at –20°C. We used a 24-well plate to culture the bacterial solution samples. The OD_600_ measurements were taken every 2 h using a microtiter plate reader.

#### A microtiter plate biofilm formation assay

We used a partially modified staining method based on those in published studies (Lee et al., 2011; Xu et al., 2015). Briefly, the overnight cultures were grown in different concentrations of DNase I (final concentrations specified above) in flat-bottomed 96-well polystyrene plates for 37°C at 24 h. For biofilm quantifications, we gently rinsed off the unadhered bacteria thrice. After drying, we stained each well with 220 µL of a 0.1% crystal violet stain for 20 min and gently washed the stain off three times with PBS. After drying, we dissolved the bound stain using 33% acetic acid for 20 min, and measured its absorbance value at OD_595_ using a microtiter plate reader.

#### Biofilm CFU assay

We used a previously described biofilm CFU assay method (Pezzoni et al., 2020). The overnight cultures were grown in 24-well plates for 24 h at 37°C, and we used the same DNase I concentrations specified above. We discarded the liquid medium with bacteria from the wells, and then washed them gently thrice. Next, we thoroughly scraped the biofilms, and determined the total number of CFUs using the serial dilution method.

#### Staining of eDNA in biofilms

We followed a published eDNA staining method (Farisa Banu et al., 2017). The overnight cultures were inoculated in the absence or presence of DNase I (final concentration = 66.7 U/L) onto 13 × 13 mm glass slides in a 24-well titer plate. After 24 h of incubation at 37 °C, we gently washed the glass slides and stained them with a 20-µM propidium iodide solution (10 mL; Shanghai Sigma-Aldrich Trading, Shanghai, China). We visualized and photographed the samples under a fluorescence microscope (Nikon eclipse Ni, Tokyo, Japan).

#### RNA extraction and RT-qPCR assay

To determine the expression levels of quorum sensing (QS) system-related genes, the bacterial strain samples were grown without or with DNase I (final concentration = 66.7 U/L). The total RNA was extracted using RNAiso Plus (Takara Holdings, Kyoto, Japan). Then, 1 µg of the total RNA was used for cDNA synthesis using the PrimeScript second strand cDNA synthesis kit (cat. RR047A, Takara). Quantitative real-time polymerase chain reaction (RT-qPCR) was performed using SYBR Green II (cat. RR047A, Takara) on a LightCycler 480 II real-time PCR system (Roche) with the specific primers listed in **Table S1**. The data were analyzed using the 2^-ΔΔCt^ method as previously described (Pfaffl, 2001).

#### Confocal laser scanning microscopy analysis

We used a previously described biofilm CLSM method (Yu et al., 2022). Briefly, biofilms were grown on confocal Petri dishes for 24 h in the absence or presence of DNase I (final concentration, 66.7 U/L). After washing the biofilms with sterile PBS, we used the fluorescent LIVE/DEAD BacLight™ bacterial viability kit L13152 (Molecular Probes, Invitrogen, USA) to detect the live and dead cells according to the manufacturer’s instructions. The images were captured and processed using Leica LAS X software, Version core 3.5.7 (Leica, Wetzlar, Germany). Biofilm viability was evaluated using the Fiji macro (ImageJ) method as previously described (Mountcastle et al., 2021). Three points were randomly selected for the examination of every sample, and biomass and average thickness were assessed using COMSTAT2.1 software.

### *In vivo* experiments

#### Animal studies

The Medical Ethics Committee of the First Affiliated Hospital of Guangxi Medical University approved all the animal experiments (NO.2022-KY-E-(079)). Forty-eight healthy New Zealand rabbits provided by the Experimental Animal Center of Guangxi Medical University, weighing 2.5 ± 0.5 kg, were used in this study.

#### Pleural empyema induction and DNase I treatment

We randomly assigned 16 rabbits to either a *P. aeruginosa* or an *S. aureus* group; next, we randomly subdivided each group into two groups (medication group and control group). The rabbit models were established as published (Zhang et al., 2020) with some modifications. Briefly, all rabbits were anesthetized with pentobarbital sodium. We placed a drainage tube in the chest cavity using a deep venous catheter package. After ensuring that the pleural cavity did not contain air, we injected 2 mL/kg of LB containing *P. aeruginosa* or TSB containing *S. aureus*. Subsequently, we injected 5 mL of DNase I (200 U) or 5 mL of PBS through the catheter. The catheter was rinsed with 2 mL PBS. After 24 h, we sacrificed the rabbits and performed autopsies. All experiments were repeated three times.

#### Pleural empyema score

Two researchers who did not participate in the experiments scored the pleural empyema by assessing the amounts of pleural effusion, purulent exudate, and pleural adhesion. We used the pleural empyema score points (0–4) to classify the severities of the pleural empyema based on a published method (Zhu et al., 2006).

#### Histopathological examination

We fixed the specimens in a 10% formaldehyde solution, embedded them in wax blocks, and cut them into slices; followed by hematoxylin and eosin (H&E) staining. Hematoxylin stains the cell nuclei blue and eosin stains the cell cytoplasm, muscle tissue, and connective tissue red. For each H&E slide, we randomly selected three visual fields for viewing and photographing under a microscope. After imaging, we measured the thickness of the pleural hyperplasia using the ImageJ software (ImageJ 1.52a, Wayne Rasband National Institutes of Health, Bethesda, USA).

#### Indwelling catheter analysis

We pulled out the indwelling catheters from the chest cavity, and washed the surfaces with sterile PBS. We cut off 3 cm from the end of each catheter and split it into half. One-half was used for crystal violet staining. The catheter was rinsed three times and a half was sheared. Then, one-half of the catheter was stained with crystal violet as described above. The other half of the catheter was placed in 2 mL of sterile PBS for CFU counts. Each catheter half was vortexed for 10 min and sonicated for 1 min to completely remove the biofilm bacterial colonies from the catheter. The solutions containing colonies from each sample were counted using a 10-times dilution method. The values were recorded as l g (CFU/mL).

#### Peptide nucleic acid fluorescence *in Situ* hybridization

We performed PNA-FISH according to previously described methods (Sladjana et al., 2009; Wei et al., 2019) using two PNA-FISH kits (AdvanDx, MA, USA), the *P. aeruginosa* kit (cat. QFGNRBC1-25) and the *S. aureus* kit (cat. KT001). Briefly, fibrinous depositions were homogenized and then placed on slides. After drying, we placed a fluorescein-labeled PNA probe in a hybridization solution, and incubated it at 55°C for 60 min. Next, we removed the coverslips and washed them in a preheated (55°C) stringent wash solution for 30 min. After that, we added 4′,6-diamidino-2-phenylindole (DAPI) for 15 min. We used a fluorescence microscope to view and photograph the images. After imaging, we used the ImageJ software to measure the red or yellow fluorescence areas.

#### Scanning electron microscopy analysis

The right pleurae of the rabbits were fixed with 2.5% glutaraldehyde solution at 4°C for 2 h. We washed the specimens with PBS for 10 min each time. Next, we applied 1% osmium tetroxide for 1 h. Subsequently, we washed the specimens with PBS thrice. The specimens were dehydrated serially using a graded ethanol series (50%, 70%, 95%, and 100%). Finally, we immersed each specimen into 100% hexamethyldisilazane, and vacuum dried it. After drying, the specimens were sprayed with an IB3 (IB5) ion-sputtering instrument and we observed them using SEM.

#### Statistical analysis

We performed all the statistical analyses using the SPSS 22.0 statistical software package (IBM Corp., Armonk, NY, USA). Quantitative variables are expressed as mean ± standard deviation (SD). Comparisons between multiple groups were conducted using one-way analysis of variance (ANOVA); subsequently, the LSD *t-*test was used for pairwise comparison. Comparisons between two independent groups were performed using the Student’s *t*-test. We considered *p-*values < 0.05 as significant.

## Results

### Growth curves

**Figure 1:**
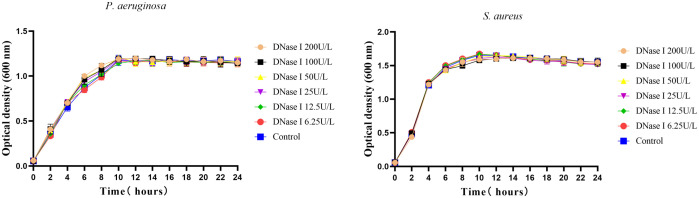
Growth curves of *P. aeruginosa* or *S. aureus* incubated with different DNase I concentrations or with drug solvent (negative control group). Three wells were set up for each group. The mean values and standard error are shown; DNase I has no effect on the growth of *P. aeruginosa* or *S. aureus*. The error bars represent the standard error of the OD600 value for each time point in the growth curves (n = 3).

To assess the effects of DNase I on bacterial growth *in vitro*, *P. aeruginosa* or *S. aureus* were grown in the presence of different dilutions of DNase I. As shown in **Figure 1**, compared with the control group, different concentrations of DNase I had no direct bactericidal and bacteriostatic effects on *P. aeruginosa* or *S. aureus* cells.

### The biofilm formation assay

**Figure 2:**
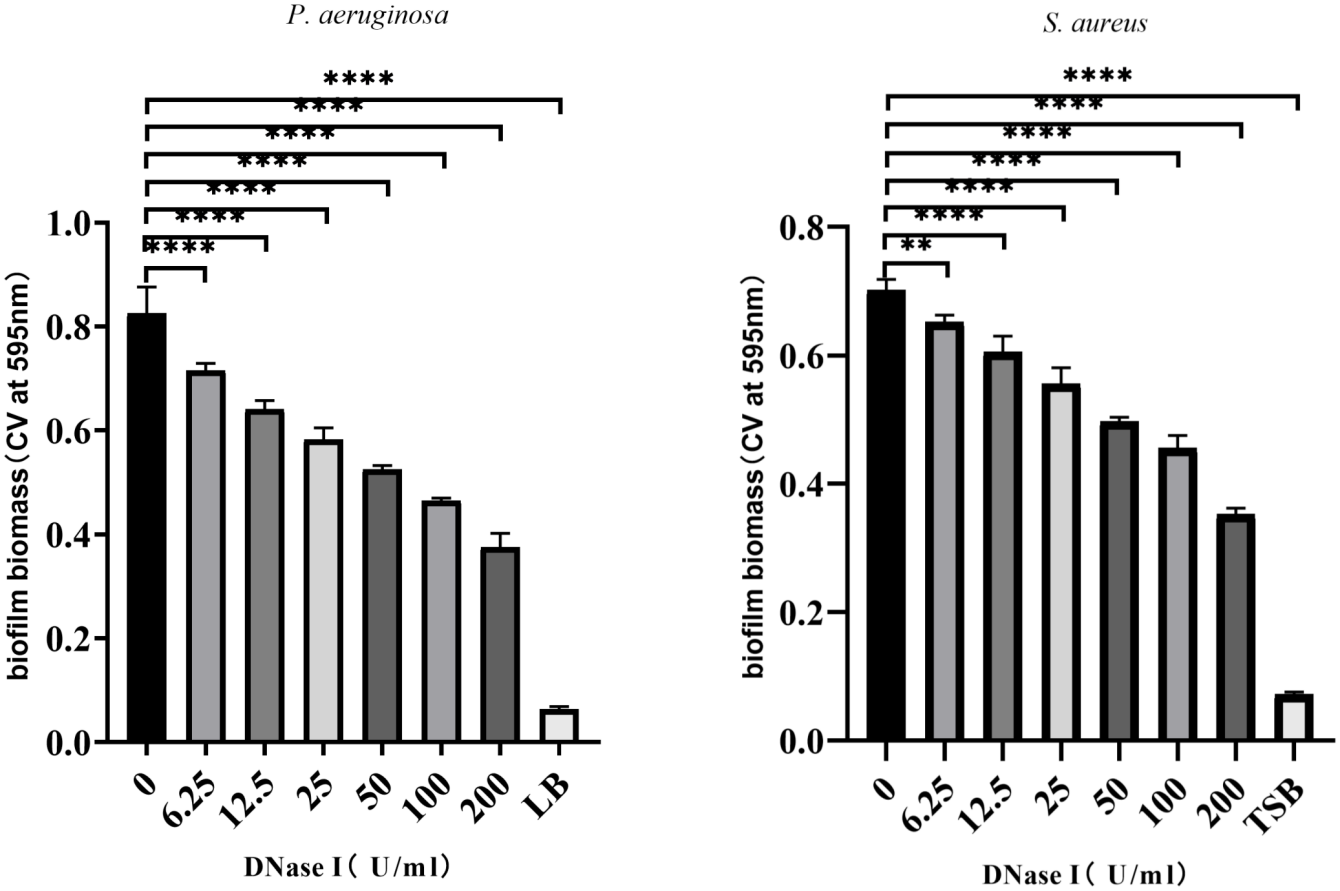
DNase I inhibition of biofilm formation by *P. aeruginosa* or *S. aureus* strains. The biofilms were stained with crystal violet and detected by OD_595_ measurements. The results are presented as mean ± SD. An asterisk indicates a significant difference, ** indicates *p* < 0.01 and **** indicates *p* < 0.0001, compared with the results in the drug-free control group using one-way ANOVA (n = 3). ANOVA tests. LB, Luria-Bertani; TSB, tryptic soy broth.

To assess the effects of DNase I on biofilm formation *in vitro*, the biofilms were stained with crystal violet, and the ODs were detected. As shown in **Figure 2**, compared with the control group, different concentrations of DNase I significantly inhibited *P. aeruginosa* or *S. aureus* biofilm formation in a dose-dependent manner (*p* < 0.05).

### Biofilm bacterial counts

**Figure 3:**
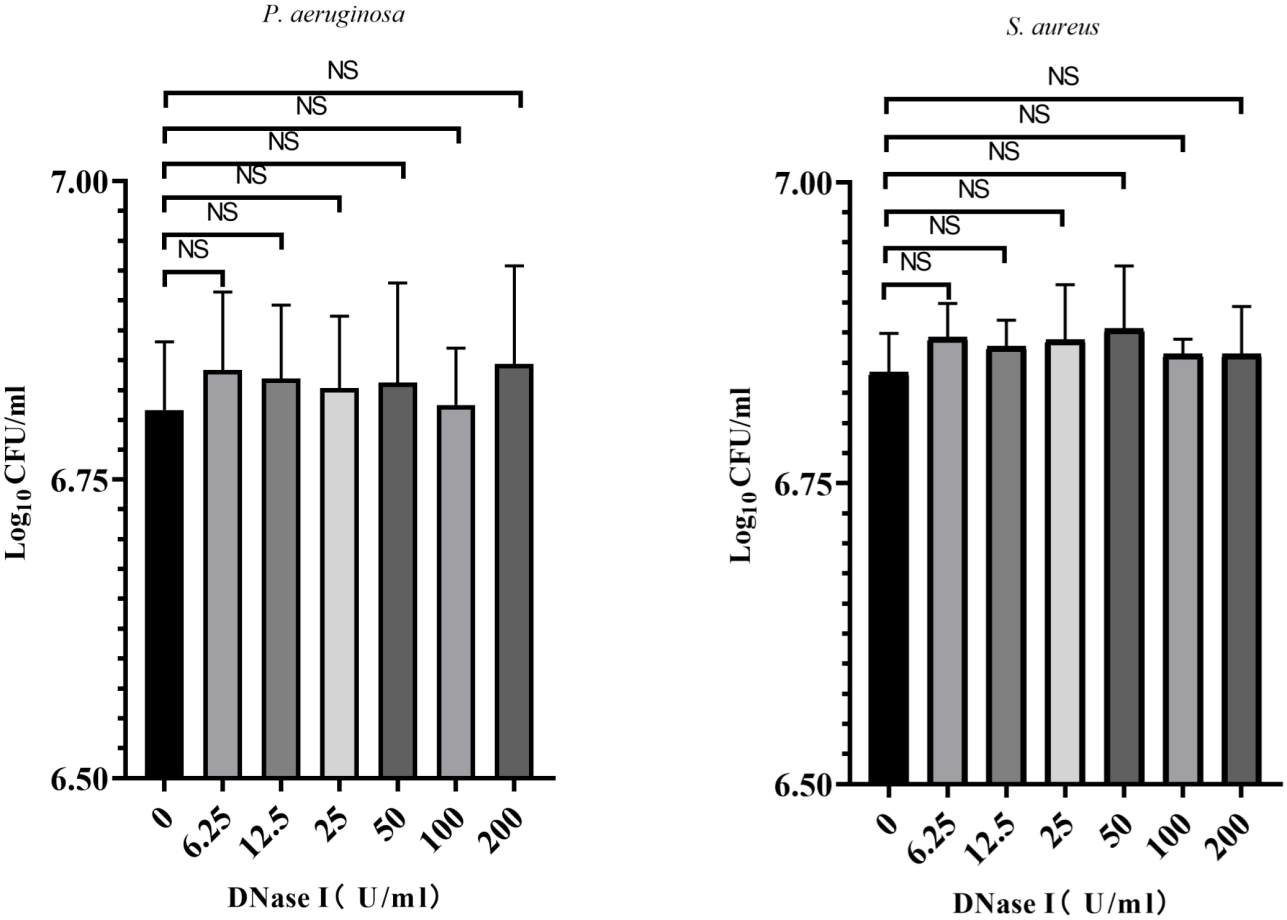
Biofilm bacterial counts were obtained after exposure to DNase I for 24 h. The results are presented as mean ± SD. NS indicates *p-*values > 0.05, compared with the results of the drug-free control group using one-way ANOVA (n = 3).

To assess the effects of DNase I on the growth of biofilm bacteria, we counted the number of biofilm bacteria exposed to different concentrations of DNase I. As shown in **Figure 3**, compared with the control group, different concentrations of DNase I had no significant effects on the number of *P. aeruginosa* or *S. aureus* in biofilms (*p* > 0.05).

### eDNA in the biofilm matrix

**Figure 4:**
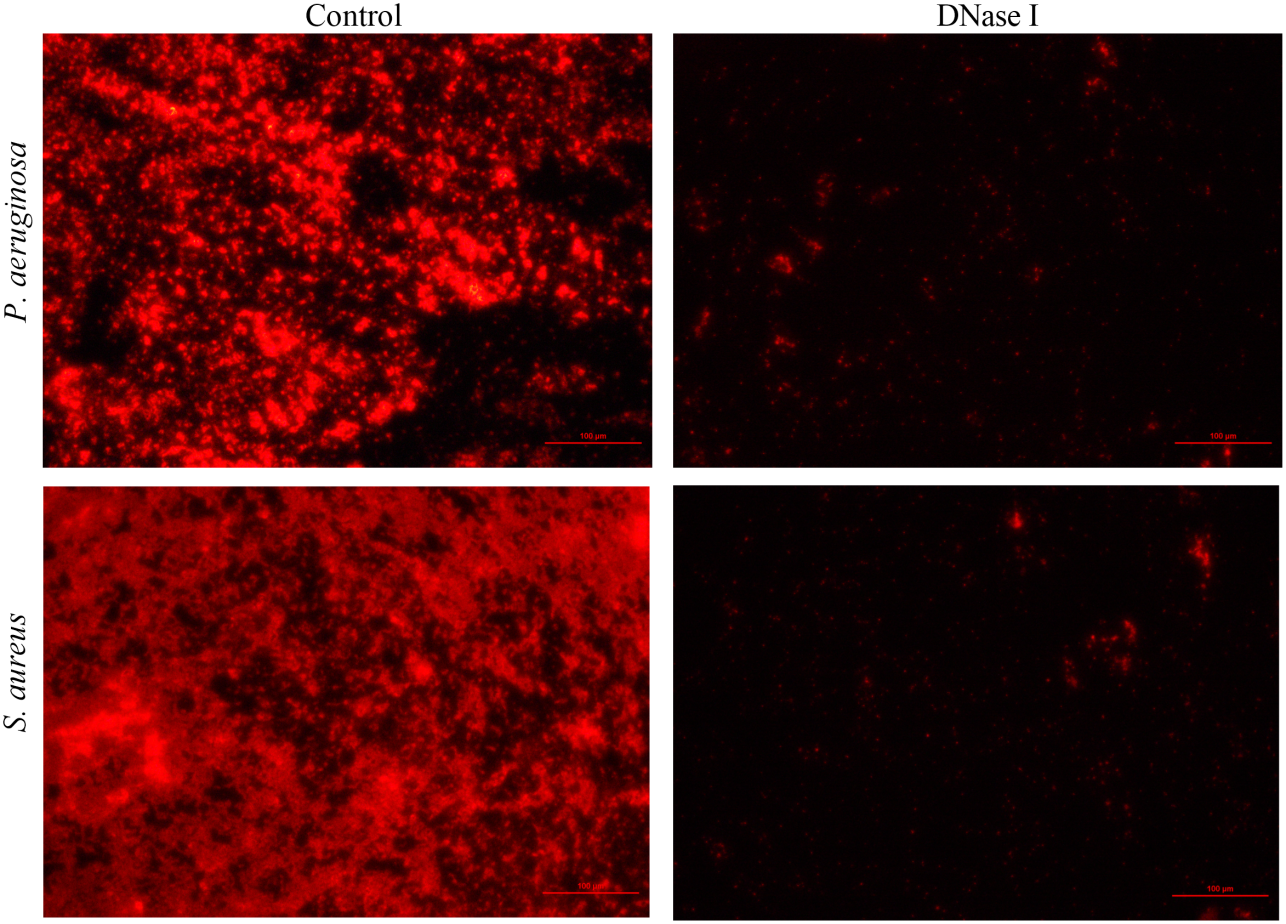
Fluorescence microscopy images of *P. aeruginosa* or *S. aureus* eDNA (100×). Propidium iodide (PI)-stained eDNA in red. In the DNase I group, the extent of PI staining decreased compared with the extent in the drug-free control group.

To detect the effect of DNase I on the eDNA of biofilm, *P. aeruginosa* or *S. aureus* biofilms were stained with propidium iodide (which specifically stains eDNA). From the fluorescent micrograph results, it was evident that the control group biofilms had more eDNA accumulation, whereas the DNase I-treated group had less eDNA accumulation (**Figure 4**).

### QS system-related gene expression

**Figure 5:**
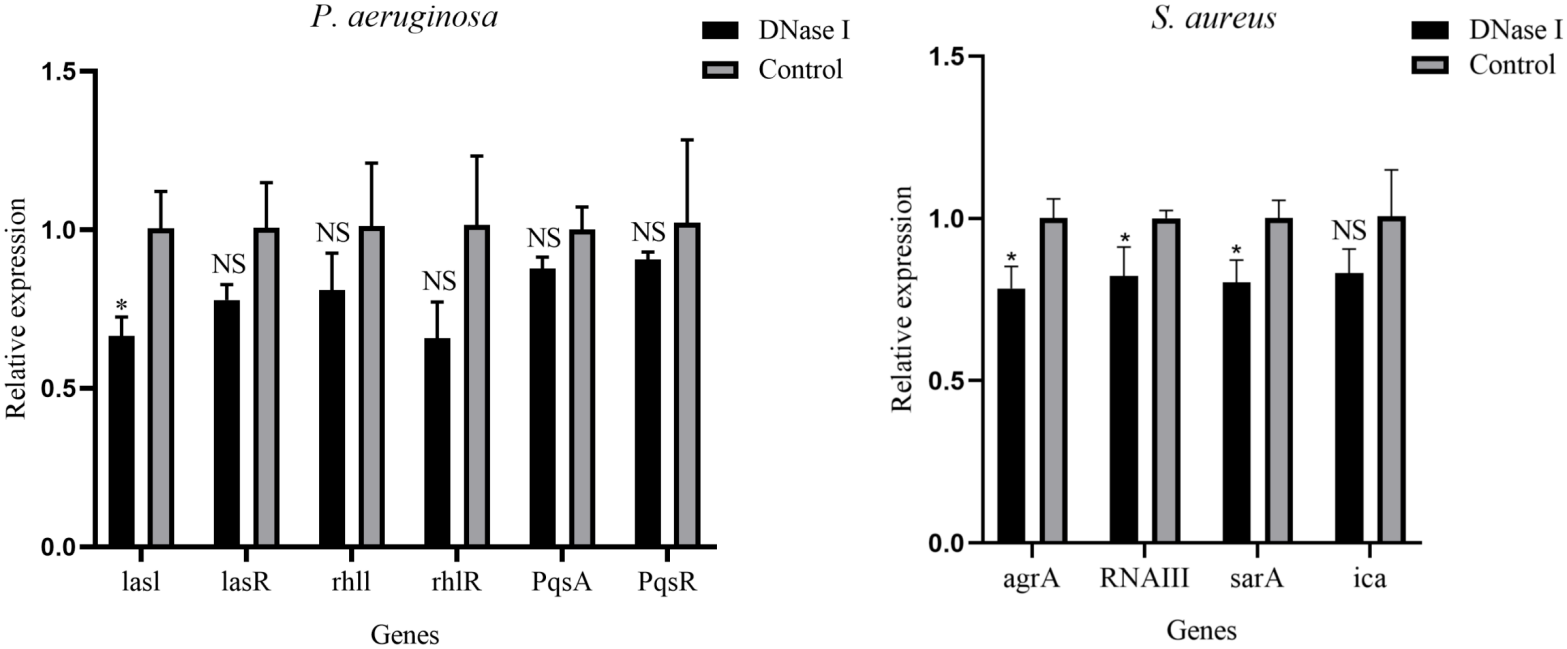
Relative expression levels of QS system-related genes of *P. aeruginosa* and *S. aureus* in the presence or absence of DNase I, as determined using real-time polymerase chain reaction. The data are presented as mean ± SD. An asterisk indicates a significant difference; * indicates *p* < 0.05 and NS indicates *p-*values > 0.05, compared with the expression values in the drug-free control group using *t*-tests (n = 3).

The RT-qPCR assay was performed to examine the effect of DNase I on the gene expression of QS systems. As shown in **Figure 5**, compared with the expression values in the untreated control group, DNase I significantly repressed the transcription level of *lasI* of *P. aeruginosa* (*p* < 0.05); DNase I repressed the transcription levels of *lasR*, *rhlI*, *rhlR*, *pqsA* and *pqsR* of *P. aeruginosa*, but they were not significant (*p* > 0.05). In addition, compared with the expression values in the untreated control group, DNase I significantly repressed the transcription levels of *agrA*, *RNAIII*, and *sarA* of *S. aureus* (*p* < 0.05); DNase I repressed the transcription level of *ica* of *S. aureus*, but it was not significant (*p* > 0.05).

### CLSM biofilm image analysis

**Figure 6:**
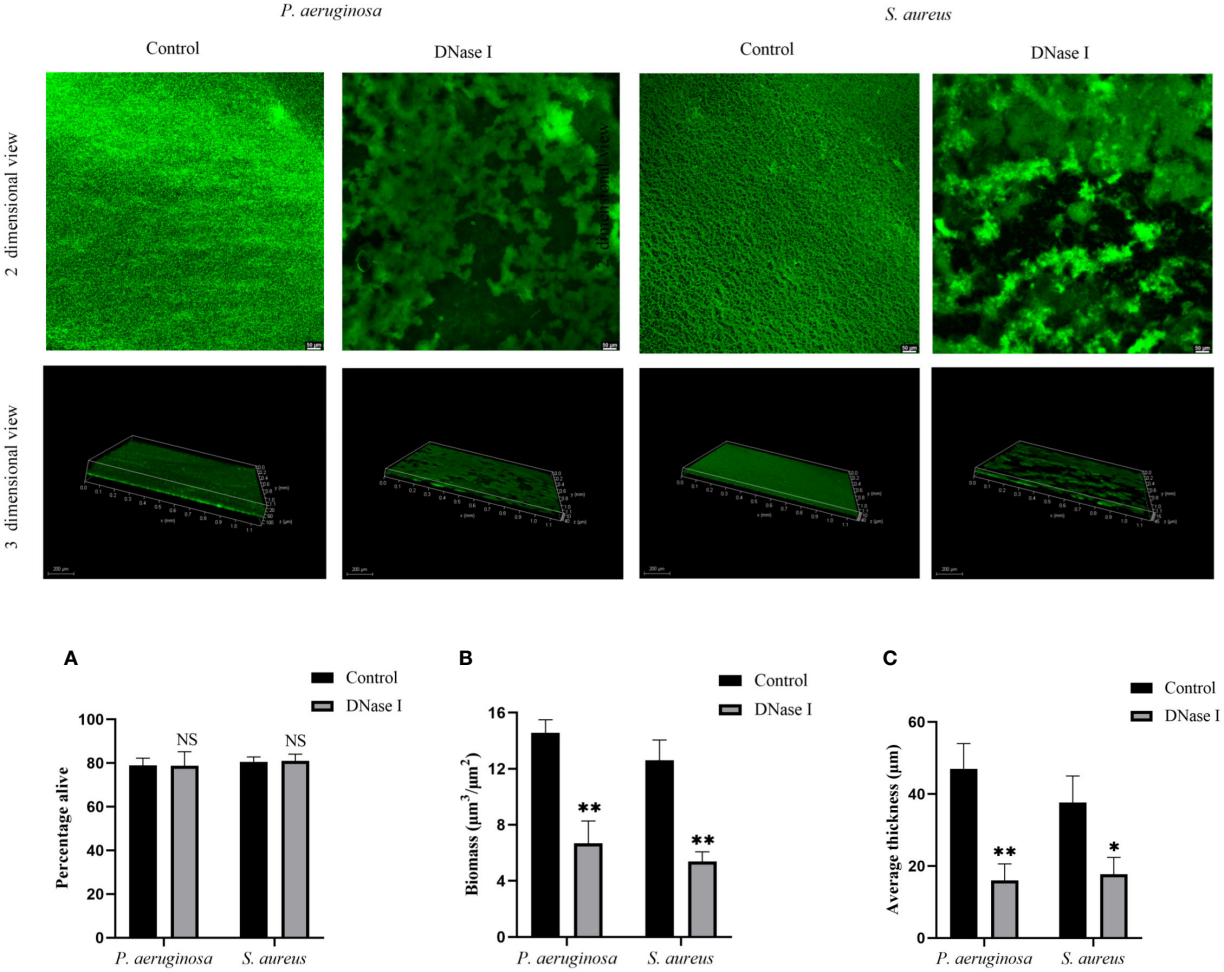
Analysis of DNase I effect on the *P. aeruginosa* and *S. aureus* biofilm structures. CLSM analysis of the biofilms formed in the presence or absence of DNase I. The bi-dimensional and three-dimensional biofilm structures were obtained using the LIVE/DEAD^®^ Biofilm Viability Kit. Viable bacteria cells with intact membranes are stained fluorescent green. **(A)** Represents the percentage of live cells. **(B)** The biofilm biomass of CLSM analysis. **(C)** Mean thickness of biofilm using CLSM analysis. The data are presented as mean ± SD. An asterisk indicates a significant difference; * indicates *p* < 0.05, ** indicates *p* < 0.01, and NS indicates *p-*values > 0.05, compared with the values in the drug-free control group using *t*-tests (n = 3).

The antibiofilm activity of DNase I on biofilm formation was also evaluated based on CLSM. As shown in **Figure 6**, the CLSM images showed a compact biofilm structure in the untreated control group, whereas the DNase I-treated group was sparse. Compared with the untreated control group, DNase I had no significant effects on the alive cells of *P. aeruginosa* or *S. aureus* in the biofilms (*p* > 0.05). To get more detailed information about the biofilm structure, we analyzed three-dimensional images. DNase I significantly decreased the biofilm biomass as compared with the untreated control group (p < 0.05). The mean biofilm thickness of the DNase I-treated group was significantly thinner than those of the untreated control group (*p* < 0.05).

### DNase I inhibits biofilm formation in rabbit empyema models

#### Macroscopic appearance and empyema scores

**Figure 7:**
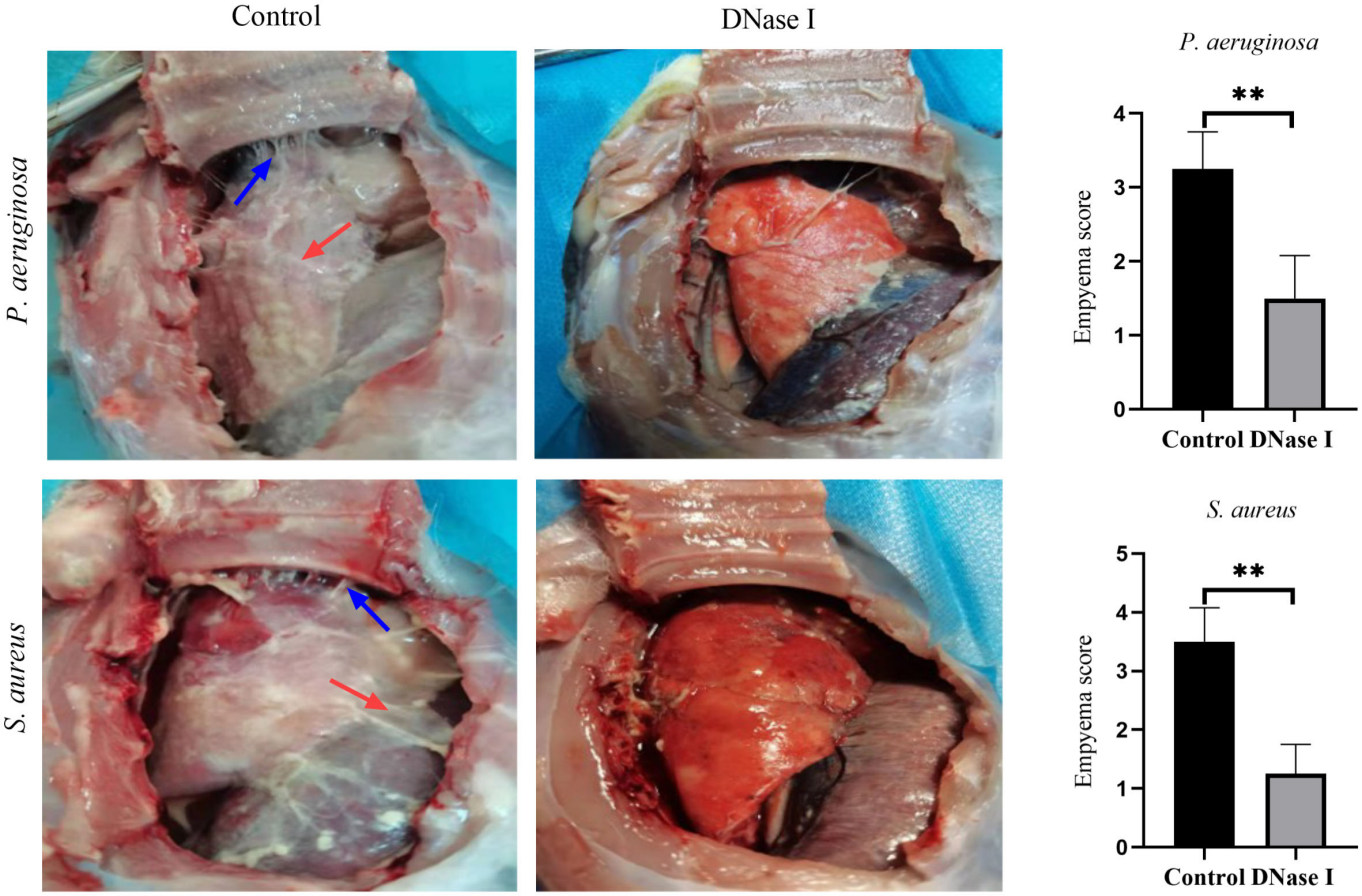
Gross pathology specimens of rabbit pleural cavities 24 h after infection and treatment. The control rabbits had more pleural adhesions and fibrin depositions between the visceral and parietal pleura than the DNase I-treated rabbits. The blue arrows indicate adhesion bands and the red arrows indicate fibrin deposition. The empyema scores are presented as mean ± SD. An asterisk indicates a significant difference; ** indicates *p* < 0.01, compared with the values in the drug-free control group using *t*-tests (n = 4).

A total of 48 rabbits were used in our experiments. No animal died in either group. Compared with the DNase I-treated rabbits, the control rabbits showed slightly reduced food and water intakes, normal defecation, lethargy, and reduced activity. On day one after infecting the rabbits with *P. aeruginosa* or *S. aureus*, the control rabbits had more fibrin deposition and more adhesive bands in the pleural cavity than the DNase I-treated rabbits; moreover, the empyema scores of the control rabbits were higher than those of the DNase I-treated rabbits (*p* < 0.05; **Figure 7**).

#### Pleural histopathology

**Figure 8:**
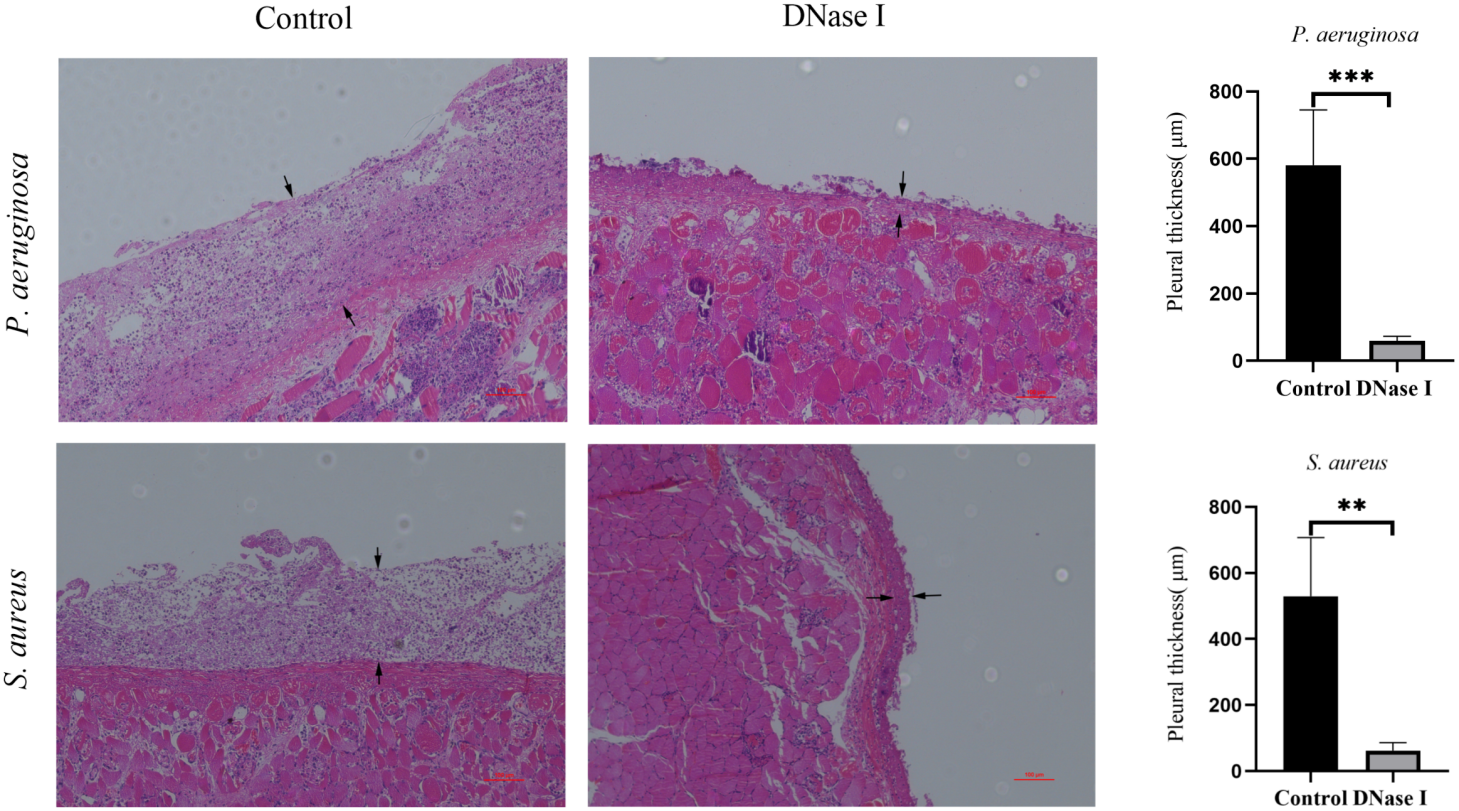
Morphological changes of the parietal pleura (H&E staining, 100×). The area between the two black arrows is the pleura. In the control rabbits, we observed parietal pleura thickening, accompanied with many inflammatory cell infiltrations. In the DNase-I treated rabbits, the parietal pleura was slightly thickened with only mild inflammation. The pleural thicknesses are presented as mean ± SD. An asterisk indicates significant difference; ** indicates *p* < 0.01 and *** indicates *p* < 0.001, compared with the values in the drug-free control group using *t*-tests (n = 4).

To assess the effects of DNase I on the pleural inflammation of empyema, we stained the parietal pleura using H&E staining. We found significant differences in the H&E-stained sections of the rabbit parietal pleura samples between the control and the DNase I- treated group. In the control rabbits, we observed varying degrees of inflammatory cell infiltrations in the parietal pleura membrane. In the DNase I-treated rabbits, we observed mild inflammatory cell infiltrations. The pleural thicknesses of the DNase I-treated rabbits were significantly thinner than those in the control rabbits (*p* < 0.05; **Figure 8**).

#### Biofilm staining on catheter tubes

**Figure 9:**
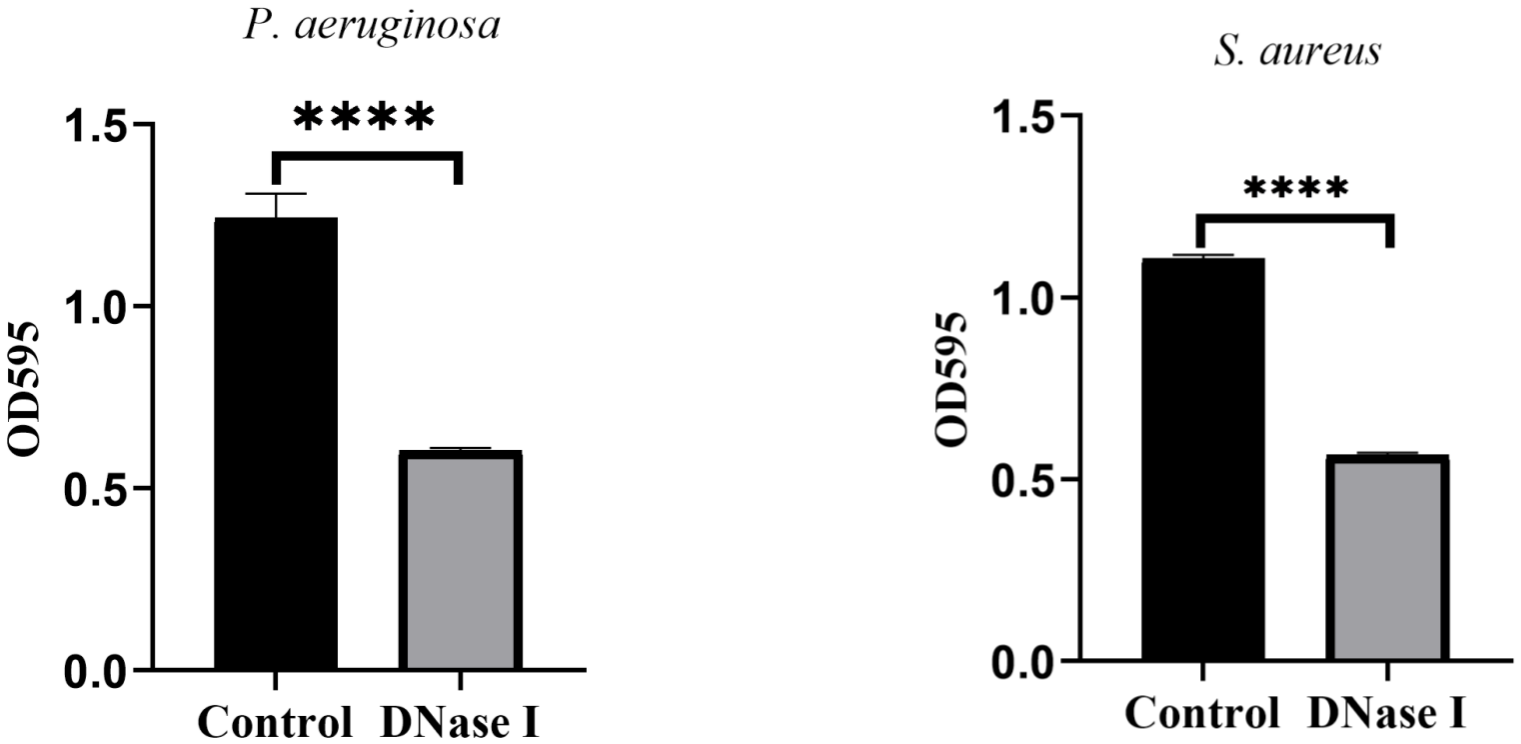
Results of the crystal violet staining of the indwelling catheters. OD values of crystal violet staining for indwelling catheters in two rabbit groups. The results are presented as mean ± SD. An asterisk indicates a significant difference; **** indicate *p* < 0.0001, compared with the values in the drug-free control group using *t*-tests (n = 4).

To assess the effects of DNase I on biofilm formation on catheter surfaces, we stained the biofilms with crystal violet and determined their OD values. As shown in **Figure 9**, the OD values of the crystal violet staining in the control rabbits were significantly higher than those in the DNase I-treated rabbits (*p* < 0.05).

#### Catheter bacteriology

**Figure 10:**
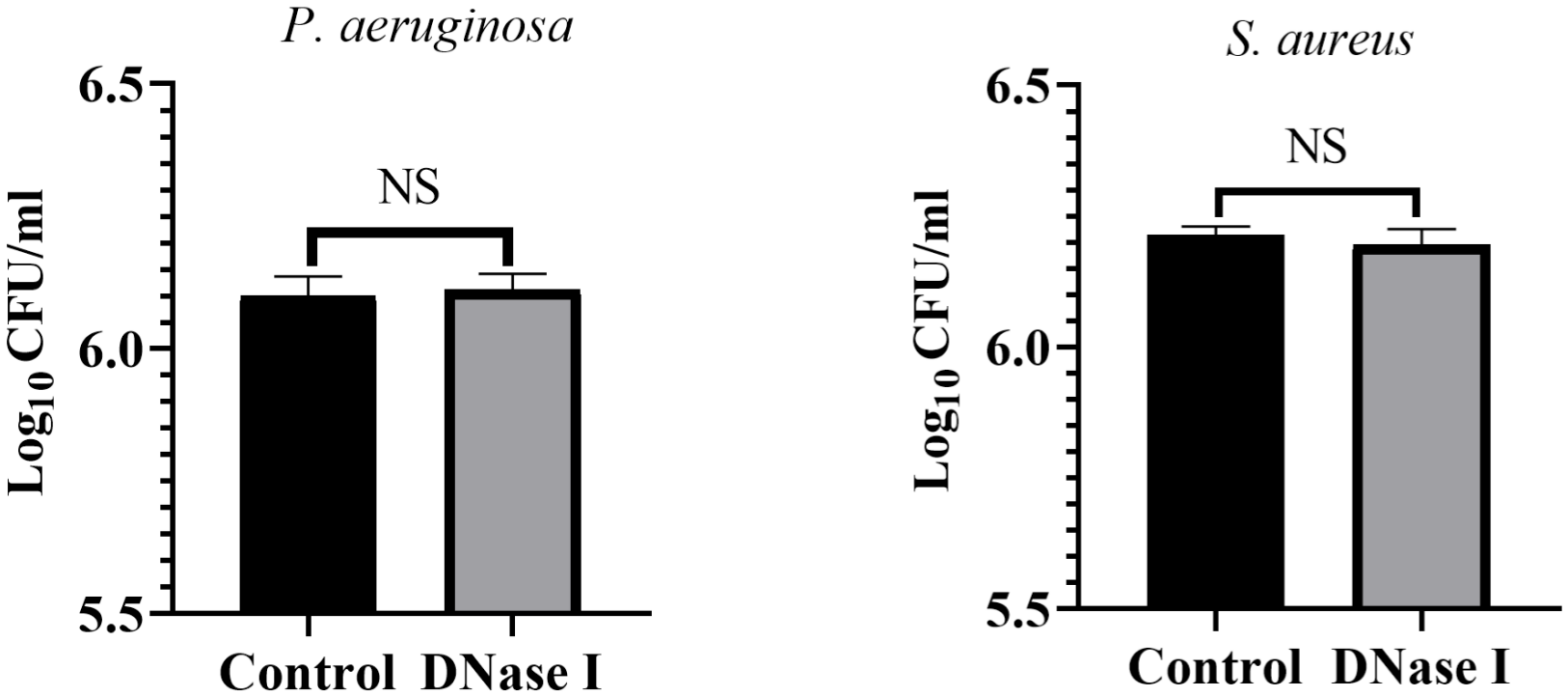
Colony counts of the indwelling catheters. Colony counts of *P. aeruginosa* or *S. aureus* on the surface of the indwelling catheters in the two rabbit groups. The results are presented as mean ± SD. NS indicates a *p* value > 0.05, compared with the values in the drug-free control group using *t*-tests (n = 4).

To assess the effects of DNase I on catheter colony counts, we diluted the catheter wash solutions and determined the count of viable colonies. **Figure 10** shows the colony counts in the control and DNase I-treated rabbits. The catheter colony counts between the control and DNase I-treated rabbits were not significantly different (*p* > 0.05).

#### Biofilms in the fibrinous deposition of purulent exudate

**Figure 11:**
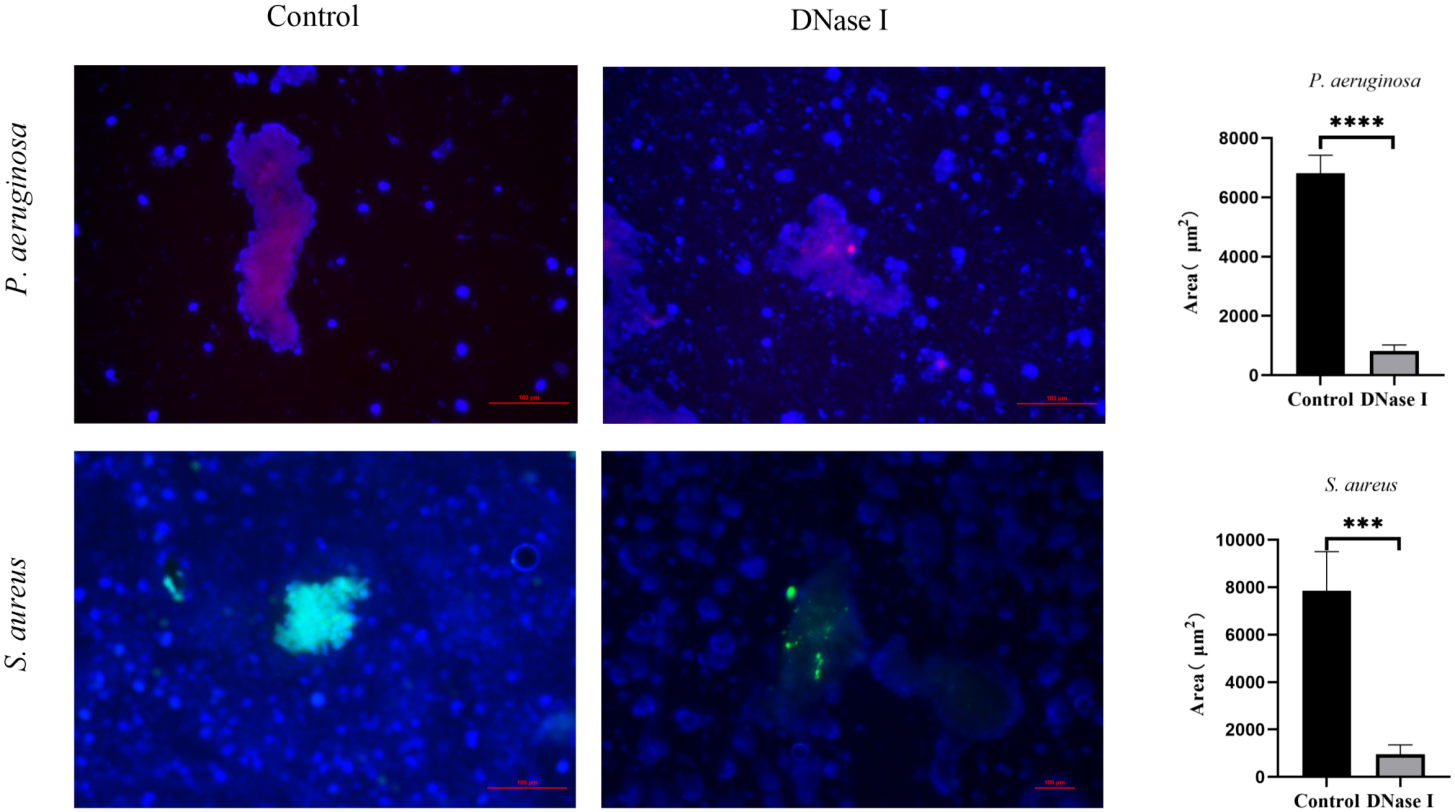
PNA-FISH of fibrinous depositions in purulent exudates (100×). The PNA-FISH kit contained a *P. aeruginosa*-specific probe (red), an *S. aureus*-specific probe (green), and an unspecific nucleic acid stain DAPI (blue) to show the biofilms. The figure shows that the mass bacteria in the control rabbits were surrounded by host cells. The red and yellow portions are the bacterial aggregates surrounded by polymorphonuclear leukocytes. In the DNase I-treated rabbits, few bacteria were surrounded by host cells. We determined the biofilm sizes using PNA-FISH. The results are presented as mean ± SD. An asterisk indicates a significant difference; *** indicates *p* < 0.001 and **** indicates *p* < 0.0001, compared with the values in the drug-free control group using *t*-tests (n = 4).

To investigate the effects of DNase I on biofilms in the fibrinous depositions of purulent exudates, we applied the PNA-FISH method to the samples to detect the ribosomal RNAs of *P. aeruginosa* or *S. aureus* in fibrinous depositions of purulent exudates. In the *P. aeruginosa* control rabbits, the bacteria (red) were surrounded by host cells and the samples showed a bright PNA-FISH signal. In the *S. aureus* control rabbits, the bacteria (yellow) were surrounded by host cells and the samples showed a bright PNA-FISH signal. These structures were aggregations. We compared the red or yellow fluorescence areas in each group of rabbits. The areas of the DNase I-treated rabbits were significantly smaller than those of the control rabbits (*p* < 0.05; **Figure 11**).

#### SEM evaluation of biofilm formation

**Figure 12:**
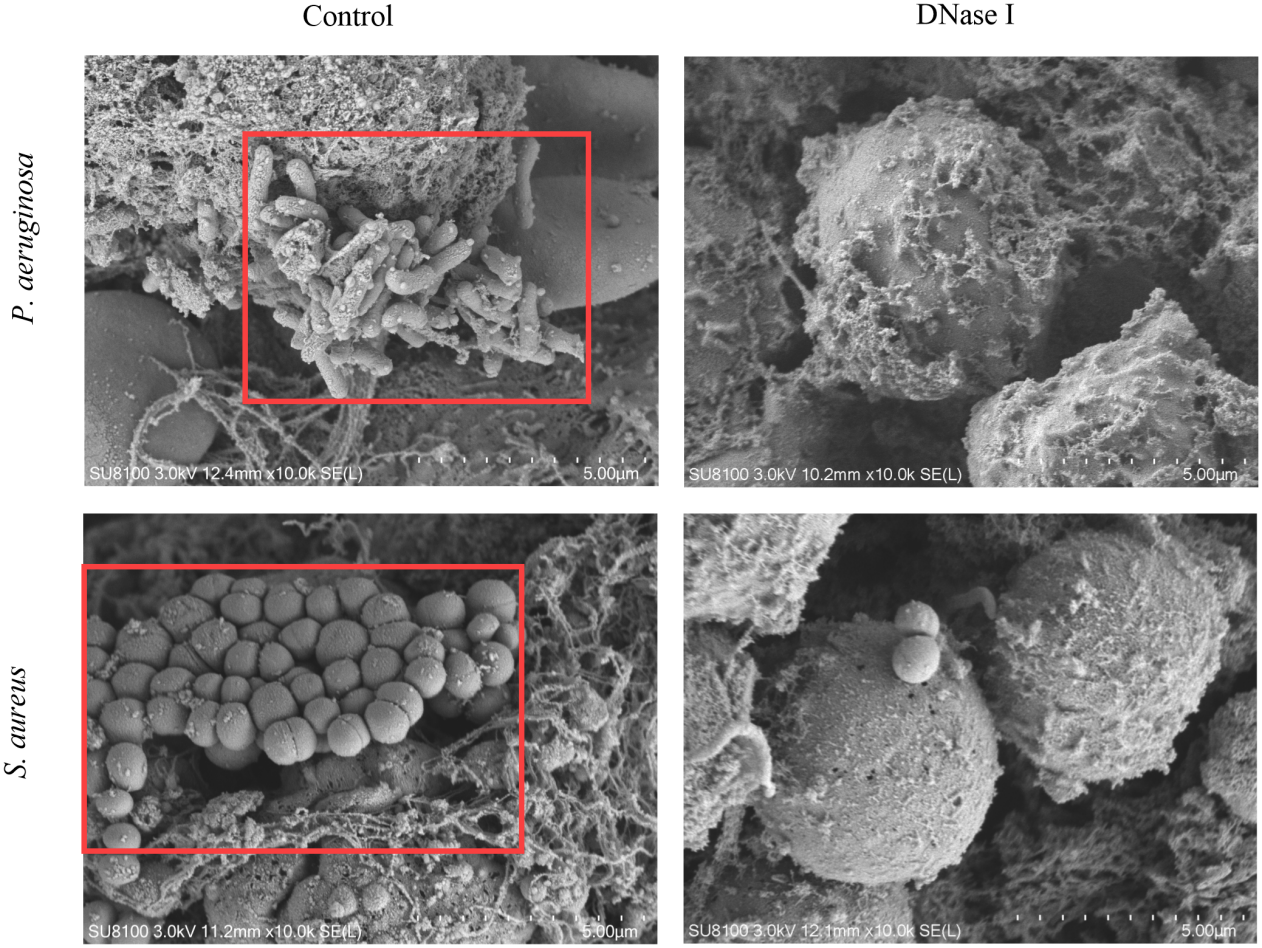
Pleural surface as seen using SEM (10,000×). In the control rabbits, we observed biofilm-like structures on the pleural surface. The *P. aeruginosa* or *S. aureus* strains were embedded in an electron-dense extracellular matrix (red box) in apparent biofilm-like structures. In the DNase-I treated rabbits, we observed only a few bacteria surrounded by a loose matrix.

To detect the effects of DNase I on biofilm structures in the pleural cavity, we applied the SEM to test samples. We observed biofilm structures on the surface of the pleural membrane formed by microorganisms adhering to the cell surfaces in the control rabbits. The microorganisms were embedded in a self-produced extracellular matrix. Conversely, these structures were absent from the surfaces of the pleural membranes in the DNase I-treated rabbits (**Figure 12**).

## Discussion

Pleural empyema is a life-threatening disease and is associated with the formation of biofilm in the pleural cavity. In a previous study, we were the first to prove that *P. aeruginosa* forms biofilms in a rabbit empyema model (Zhang et al., 2020). In this study, we assessed the effect of DNase I on the inhibition of early biofilm formation *in vitro* and *in vivo*. Our results confirmed that DNase I significantly inhibited the early biofilm formation by *P. aeruginosa* or *S. aureus*. To the best of our knowledge, this is the first study to report DNase inhibits early biofilm formation in pleural empyema.

*P. aeruginosa* and *S. aureus* cells gradually form an early biofilm from adhesion within 24 h (Tsiry et al., 2015; Jelle et al., 2022). Previous studies have shown that DNase can inhibit or degrade early-stage bacterial biofilm formation *in vitro* (Tetz et al., 2009; Kaplan et al., 2012), and that DNase was an effective treatment strategy against biofilm-associated wound infections (Kumar et al., 2019). In this study, our data reveal that DNase I inhibited early biofilm formation by *P. aeruginosa* or *S. aureus* in rabbit empyema models. The crystal violet staining results suggest that DNase I inhibited early biofilm formation by *P. aeruginosa* or *S. aureus in vitro*. More importantly, our PNA-FISH assay and SEM analysis results reveal that DNase I inhibited early biofilm formation in *P. aeruginosa*- or *S. aureus*-induced rabbit empyema models. This finding suggests that the mode of action of intrapleural DNase therapy may be to inhibit early biofilm formation in pleural empyema.

eDNA is known to be important for biofilm stability, and DNase treatment can remove eDNA from biofilms, particularly during bacterial attachment and the initial stages of biofilm formation (Panlilio and Rice, 2021). Cleavage of eDNA by DNase can promote the penetration of antibiotic molecules and decrease biofilm biomass (Liu et al., 2020). Banu et al. (Banu et al., 2017) found that DNase released eDNA and inhibited *P. aeruginosa* biofilm formation. Our eDNA staining results show that DNase I degraded the eDNA in the biofilm matrix, which was consistent with the results of previous studies (Farisa Banu et al., 2017; Saharut et al., 2020). Thus, these suggest that DNase inhibits early biofilm formation by degrading the eDNA of the extracellular matrix. However, a recent study found that eDNA only localizes outside the matrix rather than inside the biofilm in a murine model infected by *P. aeruginosa* (Alhede et al., 2020). The role of eDNA in pleural empyema remains unclear, and we will further investigate this in animal models in the future.

The effect of DNase I on biofilm formation by *P. aeruginosa* or *S. aureus* was further evaluated by observing and analyzing of biofilm using CLSM. The surface morphology and three-dimensional structure of the biofilms could be observed using CLSM (Luo et al., 2018), and CLSM coupled with live/dead fluorescent staining is a powerful tool for the analysis of biofilm structure (Lacotte et al., 2022). The Fiji macro analysis method was used in ImageJ to calculate CLSM bacterial viability, which is an automated image analysis technique that has shown reliable measurements of biomass and cell viability (Mountcastle et al., 2021). Many bespoke software, such as Imaris (Chávez de Paz et al., 2008), COMSTAT (Yin et al., 2022), PHLIP (Lukas et al., 2006), and BiofilmQ (Hartmann et al., 2021), have been used to quantitatively analyze confocal images. Currently, the most widely used biofilm image analysis software in the literature is COMSTAT (Heydorn et al., 2000), which provides the tool to objectively determine the differences in biofilm morphology and parameters for 3D phenotyping. A previous study found out, through CLSM observation and COMSTAT analysis, that DNase reduced the biofilm biomass and average thickness of *P. aeruginosa* biofilm (Farisa Banu et al., 2017), which is consistent with our results. Our COMSTAT analysis results show that DNase I reduced the biomass and average thickness of *P. aeruginosa* or *S. aureus* biofilm. Thus, our CLSM results further confirmed that DNase I inhibited early biofilm formation by *P. aeruginosa* or *S. aureus in vitro*.

Our data also show that several QS system-related genes were downregulated upon addition of DNase I to the biofilm formation. Biofilm formation is a complex and unique dynamic process, and the QS system plays an important role in the regulation of genes that control biofilm formation. Chen et al. (Chen et al., 2016) proved that baicalein inhibited *S. aureus* biofilms by downregulating the QS system-associated genes *agrA*, *RNAIII*, *sarA*, and *ica*. Coelho et al. (Coelho et al., 2021) demonstrated that pyranoanthocyanins inhibited biofilm formation and interfered with the expression of the QS system-related genes in *P. aeruginosa* and *S. aureus.* However, there is a lack of research concerning the molecular mechanisms of DNase inhibition in biofilm formation. Our results reveal that the expression levels of several QS system-related genes (*lasR*, *rhlI*, *rhlR*, *pqsA*, *pqsR*, and *ica*) were downregulated after DNase I treatment, but it was not significant. A possible explanation is that biofilm formation is a complex process regulated by multiple pathways, such as the cyclic diguanosine monophosphate, QS system, and *Pseudomonas* quinolone signal. DNase may inhibit biofilm formation through multiple pathways. Our results suggest that one of the mechanisms by which DNase I inhibited biofilm formation by regulating the genes related to the QS-system *P. aeruginosa* and *S. aureus*. The specific mechanisms by which DNase inhibits biofilm formation need to be further studied and discussed.

Biofilms are composed of bacterial communities surrounded by a self-produced polymer matrix that protects them from diverse environmental stresses (Nourbakhsh et al., 2022). eDNA is an important component of the biofilm extracellular matrix (Rajendran et al., 2013), which exists in two forms, namely B-form eDNA (B-DNA) and Z-form eDNA (Z-DNA). B-DNA is found mostly in early biofilms and is sensitive to DNase, but B-DNA turns into Z-DNA in mature biofilms, and this form is resistant to DNase (Buzzo et al., 2021). DNase typically has no apparent effects on mature biofilms (*i.e.*, those growing for > 24 h) (Koo et al., 2017). In addition, studies (Schlafer et al., 2018; Sharma and Pagedar Singh, 2018) have found that DNase is effective against the formation of early biofilms within 24 h, but not after that. Therefore, for this study, we only focused on the early biofilms within 24 h of infection. For mature biofilms (*i.e*., those growing for 36 h), or earlier biofilms (*i.e*., those growing for 12 h), we will study the processes of pleural biofilms using multiomics, and to clarify the development of biofilm in different periods in pleural empyema in the future.

Bacterial growth and colony formation are important factors in biofilm formation. Conversely, the biofilms support bacterial growth and protect them from environmental stress. In our study, we found that DNase I had no effect on *P. aeruginosa* or *S. aureus* biofilm bacterial counts *in vitro* and *in vivo*, which is in agreement with results from previous studies (Kaplan et al., 2012; Xu et al., 2015). Karygianni *et al*. reported that DNase affects the structural integrity and spatial distribution of biofilms, and that it has no effect on the total oral bacteria CFUs (Karygianni et al., 2021). This indicates that DNase acts as an antibiofilm agent, not as an antimicrobial; moreover, clinical experience shows that DNase needs to be combined with antibiotics in the treatment of pleural empyema.

In our study, we found that DNase I decreased the empyema score and it was effective for treating rabbit empyema. Zhu et al. established a rabbit empyema model infected by *Pasteurella multicoda* and compared the effect of tPA and rhDNase in the treatment of empyema in rabbits (Zhu et al., 2006). Their study showed that DNase alone was ineffective in decreasing the empyema scores and in treating rabbit empyema. Their results disagree with our experimental results. A possible explanation for this difference is that the antibiofilm effect of DNase decreased with the advancing age of the biofilm; Zhu et al. administered DNase after the diagnosis of empyema, which may be treated because of the mature biofilms; however, DNase I was used for early biofilm in our study. In addition, the complex environment of the chest cavity may also affect the efficacy of DNase. Under the influence of confounding factors, such as pH values and oxygen concentrations in the pleural cavity, the DNase enzymatic activity may vary widely.

For the treatment of pleural empyema, antibiotics and drainage are the first-line therapy (Addala et al., 2021). However, the structure of biofilms better protects bacteria from host defense systems (Krychowiak et al., 2014). In addition, the biofilms seem to enhance the pathogen’s resistance to antibiotics, and unfortunately, the antibiotics we generally use cannot penetrate biofilms to kill bacteria (Hall and Mah, 2017; Wang et al., 2020). Microbial cells within biofilms are more resistant to antibiotics than planktonic cells, which are difficult to remove completely, leading to the emergence of bacterial infections caused by pathogens, such as multidrug-resistant, extensively drug-resistant, and totally drug-resistant bacteria. This is a major cause of persistent inflammation and difficulties in treating chronic infections. Irregular diagnoses and treatments in the clinic may lead to empyema developing into chronic infections. Thus, antibiofilm agents are an option in the management of pleural empyema. Without protection from biofilms, the infecting bacteria may be more exposed to the host immune cells and more easily eliminated (Shang et al., 2022). We found that DNase I inhibited biofilm formation in rabbit empyema models, suggesting that DNase is a potential antibiofilm agent for the treatment of pleural empyema.

Despite continuous progress, understanding of the pathogenesis and management of pleural empyema remains insufficient. In our study, we demonstrated that DNase I inhibited biofilm formation in rabbit empyema models. To some extent, this makes clinical sense. Our PNA-FISH analysis proves that DNase I treatment reduced bacterial aggregation to form biofilms, indicating that DNase may be used for the effective prevention and treatment of chronic empyema infection caused by biofilms. More importantly, DNase inhibits biofilms, which can increase the sensitivity of pathogens to antibiotics, and thus DNase may be used to reduce the use of antibiotics and for the development of resistant bacteria. In addition, intrapleural therapy with DNase does not only reduce the viscosity of the pleural fluid, but also plays the role of an antibiofilm, and thus DNase may be used to improve the removal of bacteria from the pleural cavity. Meanwhile, in a clinical trial, DNase monotherapy was associated with an increase in surgical referrals and provided no fluid-drainage benefit (Rahman et al., 2011); therefore, DNase is recommended with a fibrinolytic agent for adult patients with pleural empyema. However, the optimal dose, duration and timing of DNase treatment for empyema are still not determined, and further studies are needed to elucidate these.

Our study has limitations that should be considered. First, we only observed monotherapy with DNase in the treatment of pleural empyema biofilms. Second, the rabbit sample size of our study was small. Third, although the study provided evidence that DNase was effective to inhibit biofilm formation, we did not investigate DNase therapy on mature biofilms. Finally, empyema is caused by multi-species pathogens, such as *S. aureus, P. multocida, A. balticum, B. pseudomallei*, and *P. aeruginosa*. Our experiment only focused on a single pathogen in rabbit empyema models, and did not explore mixed pathogen infection. We will investigate mixed pathogen infection of biofilms and mixed pathogen interaction in animal empyema models in the future.

In conclusion, to the best of our knowledge, our study is the first to investigate the inhibition of early biofilm formation in rabbit empyema models by DNase. The results show that DNase significantly inhibited *P. aeruginosa* or *S. aureus* early biofilm formation *in vitro* and *in vivo*. Therefore, DNase inhibits early biofilm formation in *P. aeruginosa*- or *S. aureus*-induced rabbit empyema models and shows its therapeutic potential against empyema biofilm. These results provide a theoretical foundation for the pathogenesis and management of pleural empyema.

## Data availability statement

The raw data supporting the conclusions of this article will be made available by the authors, without undue reservation.

## Ethics statement

The animal study was reviewed and approved by the Medical Ethics Committee of the First Affiliated Hospital of Guangxi Medical University [NO.2022-KY-E-(079)].

## Author contributions

WD, YL and XT conducted the experiments, analyzed the data, and wrote the manuscript. DL and JL participated in the experiments and writing. JL, LL, WZ and LY analyzed the data. KW, JK and ZC designed the study and participated in data analysis and discussion. All authors read and approved the final version of the manuscript for submission.

## Funding

This work was supported by the National Natural Science Foundation of China under Grant (No. 82260023, 81760024, 82160783 and 82104499); the Natural Science Foundation of Guangxi Province (No. 2022GXNSFAA035646); the Key Research Program of Guangxi Science and Technology Department (No. AB21196010); the Advanced Innovation Teams and Xinghu Scholars Program of Guangxi Medical University; the Medical Excellence Award Funded by the Creative Research Development Grant from the First Affiliated Hospital of Guangxi Medical University (No. XK2019025); and Partially Supported by Open Funding Project of State Key Laboratory of Microbial Metabolism Of Shanghai Jiao Tong University (No. MMLKF22-06); The Clinical Research Climbing Program Youth Science and Technology Morning Star Program of the First Affiliated Hospital of Guangxi Medical University (No. 201903032 and YYZS2020016).

## Conflict of interest

The authors declare that the research was conducted in the absence of any commercial or financial relationships that could be construed as a potential conflict of interest.

## Publisher’s note

All claims expressed in this article are solely those of the authors and do not necessarily represent those of their affiliated organizations, or those of the publisher, the editors and the reviewers. Any product that may be evaluated in this article, or claim that may be made by its manufacturer, is not guaranteed or endorsed by the publisher.
