# Supplementary material for: DNase inhibits early biofilm formation in Pseudomonas aeruginosa- or Staphylococcus aureus-induced empyema models
Source: Front Cell Infect Microbiol. 2022 Oct 12;12:917038. doi: 10.3389/fcimb.2022.917038 (PMC9597695; doi:10.3389/fcimb.2022.917038)
Supplement: Supplementary file 1 [file DataSheet_1.docx]

**S1 Table. Primers used in *P. aeruginosa* RT-qPCR analysis.** ^[1]^

| Target gene | Type | Primer sequences |  |
| --- | --- | --- | --- |
| 16S rRNA (PA) | Fw | 5’-CGTCCGGAAACGGCCGCT-3’ |  |
|  | Rev | 5’-CTCTCAGACCAGTTACGG-3’ |  |
| *lasI* | Fw | 5’-AGGCGTGGAGAAGATGATG-3’ |  |
|  | Rev | 5’-ATCTGGGTCTTGGCATTGAG-3’ |  |
| *lasR* | Fw | 5’-GTGGAAAATTGGAGTGGAGCG-3’ |  |
|  | Rev | 5’-GTAGTTGCCGACGATGAAGG-3’ |  |
| *rhlI* | Fw | 5’-TGCAGCCATTCCGGGTGGTA-3’ |  |
|  | Rev | 5’-TCGTTCGCAACGGCGTGAT-3’ |  |
| *rhlR* | Fw | 5’-GGGTTGGACATCAGCATCGG-3’ |  |
|  | Rev | 5’-TTGCTCAGCGTGCTTTCCGT-3’ |  |
| *PqsA* | Fw | 5’-GACCGGCTGTATTCGATTC-3’ |  |
|  | Rev | 5’-GCTGAACCAGGGAAAGAAC-3’ |  |
| *PqsR* | Fw | 5’-CTGATCTGCCGGTAATTGG-3’ |  |
|  | Rev | 5’-ATCGACGAGGAACTGAAGA-3’ |  |

**S2 Table. Primers used in *S. aureus* RT-qPCR analysis.** ^[2]^

| Target gene | | Type | Primer sequences | |  |
| --- | --- | --- | --- | --- | --- |
| 16S rRNA (SA) | | Fw | | 5’-CCATAAAGTTGTTCTCAGTT-3’ |  |
|  |  | Rev | | 5’-CATGTCGATCTACGATTACT-3’ |  |
| *agrA* | | Fw | | 5’-ACGTGGCAGTAATTCAGTGTATGTT-3’ |  |
|  |  | Rev | | 5’-GGCAATGAGTCTGTGAGATTTTGT-3’ |  |
| *sarA* | | Fw | | 5’-GCTGTATTGACATACATCAGCGAAA-3’ |  |
|  |  | Rev | | 5’-CGTTGTTTGCTTCAGTGATTCGT-3’ |  |
| *RNAIII* | | Fw | | 5’-GAATTTGTTCACTGTGTCGATAATCCATTT-3’ |  |
|  |  | Rev | | 5’-GAAGGAGTGATTTCAATGGCACAAGATAT-3’ |  |
| *ica* | | Fw | | 5’-TCGCACTCTTTATTGATAGTCGCTACGAG-3’ |  |
|  |  | Rev | | 5’-TGCGACAAGAACTACTGCTGCGTTAAT-3’ |  |
|  |  |  |  |  |  |

**References**

1. Luo J, Dong B, Wang K, et al. Baicalin inhibits biofilm formation, attenuates the quorum sensing-controlled virulence and enhances Pseudomonas aeruginosa clearance in a mouse peritoneal implant infection model. PLoS One*.*2017, 12(4):e0176883.

2. Chen Y, Liu T, Wang K, et al. Baicalein Inhibits Staphylococcus aureus Biofilm Formation and the Quorum Sensing System In Vitro. PLoS One*.*2016, 11(4):e0153468.
